# Physiology, fast and slow: bacterial response to variable resource stoichiometry and dilution rate

**DOI:** 10.1128/msystems.00770-24

**Published:** 2024-07-09

**Authors:** Logan M. Peoples, Jana Isanta-Navarro, Benedicta Bras, Brian K. Hand, Frank Rosenzweig, James J. Elser, Matthew J. Church

**Affiliations:** 1 Flathead Lake Biological Station, University of Montana, Polson, Montana, USA; 2 Department of Biology, University of Copenhagen, Copenhagen, Denmark; 3 Division of Biological Sciences, University of Montana, Missoula, Montana, USA; 4 School of Biological Sciences, Georgia Institute of Technology, Atlanta, Georgia, USA; Universiteit Leiden, Leiden, Netherlands

**Keywords:** resource limitation, growth rate, bacteria, transcriptomics, stoichiometry, *Pseudomonas putida*

## Abstract

Microorganisms grow despite imbalances in the availability of nutrients and energy. The biochemical and elemental adjustments that bacteria employ to sustain growth when these resources are suboptimal are not well understood. We assessed how *Pseudomonas putida* KT2440 adjusts its physiology at differing dilution rates (to approximate growth rates) in response to carbon (C), nitrogen (N), and phosphorus (P) stress using chemostats. Cellular elemental and biomolecular pools were variable in response to different limiting resources at a slow dilution rate of 0.12 h^−1^, but these pools were more similar across treatments at a faster rate of 0.48 h^−1^. At slow dilution rates, limitation by P and C appeared to alter cell growth efficiencies as reflected by changes in cellular C quotas and rates of oxygen consumption, both of which were highest under P- and lowest under C- stress. Underlying these phenotypic changes was differential gene expression of terminal oxidases used for ATP generation that allows for increased energy generation efficiency. In all treatments under fast dilution rates, KT2440 formed aggregates and biofilms, a physiological response that hindered an accurate assessment of growth rate, but which could serve as a mechanism that allows cells to remain in conditions where growth is favorable. Our findings highlight the ways that microorganisms dynamically adjust their physiology under different resource supply conditions, with distinct mechanisms depending on the limiting resource at slow growth and convergence toward an aggregative phenotype with similar compositions under conditions that attempt to force fast growth.

**IMPORTANCE:**

All organisms experience suboptimal growth conditions due to low nutrient and energy availability. Their ability to survive and reproduce under such conditions determines their evolutionary fitness. By imposing suboptimal resource ratios under different dilution rates on the model organism *Pseudomonas putida* KT2440, we show that this bacterium dynamically adjusts its elemental composition, morphology, pools of biomolecules, and levels of gene expression. By examining the ability of bacteria to respond to C:N:P imbalance, we can begin to understand how stoichiometric flexibility manifests at the cellular level and impacts the flow of energy and elements through ecosystems.

## INTRODUCTION

Stoichiometric imbalances occur in microorganisms when the proportions of elements available for their growth (e.g., Carbon:Nitrogen:Phosphorus; C:N:P) differ from those optimal for biomass synthesis and metabolism. Such imbalances can have consequences for the flow of elements and energy through ecosystems, potentially lowering trophic efficiencies and influencing nutrient cycling (1, 2). Deviations in the proportions or concentrations of growth-requiring elements relative to cellular demands can result in energy or nutrient limitation of population sizes (*Liebig* limitation) or growth rates (*Blackman* limitation) (3–5). Such resource (energy and nutrient) limitations can cause dramatic alterations in organismal physiology, including changes in elemental stoichiometry, biochemical composition, and gene transcription (6, 7). Here, we use the more general term *stress* to describe the pressure imposed by imbalanced resource ratios while making no inherent assumptions about changes in physiology.

The physiology of an organism is dependent on its rate of growth. The tipping point that defines the transition between when one resource becomes limiting relative to another can vary with growth rate (8). At slow growth rates, heterotrophic bacteria vary their biomass stoichiometry in response to changes in resource stoichiometry, including C:N:P ratios and macromolecular composition (9–11). By contrast, when organisms approach their maximal growth rates (μ_max_), biomass stoichiometry shows less variability despite differences in resource supply ratios (12–14). To explain such observations, Monod reasoned that as an organism approaches its maximum growth rate, all cellular reactions operate at some optimal rate, leading to convergence in the composition of cellular elemental and macromolecular pools (15). However, the mechanisms underlying how bacteria modulate cellular physiology in response to both resource stress and growth rate are not well explored.

The genus *Pseudomonas* is taxonomically diverse, near ubiquitous in its distribution, and remarkable for its capacity to adjust to different environments (16–18). One of the most well-studied pseudomonads is *Pseudomonas putida* KT2440 (19, 20). KT2440 is known for its broad metabolic versatility and genetic plasticity, generating interest in its ability to cope with environmental stress as well as its biotechnological potential (21, 22). The physiology of this organism has been thoroughly studied, including through the creation of whole-genome sequences and metabolic models (23–25). KT2440 responds dynamically to nutrient limitation through changes in gene expression and energy storage (26–28). Moreover, under nutrient-replete conditions that promote rapid growth, *P. putida* can form biofilms that subsequently disperse at the onset of nutrient starvation (29–31). To date, less is known about the mechanisms underlying how KT2440 responds to the interplay between resource stoichiometry and growth rate.

Here, we used *P. putida* KT2440 as an experimental model to gain insight into the flexibility of cell physiology in the face of resource stress. We asked (i) How does variable resource stoichiometry impact biomass stoichiometry and metabolic activity? and (ii) How does bacterial physiology change as a function of growth rate? To answer these questions, we cultured KT2440 in chemostats at both relatively slow (0.12 h^−1^) and fast (0.48 h^−1^) dilution rates with media resource stoichiometry designed to facilitate balanced growth or to promote carbon (C), nitrogen (N), or phosphorus (P) stress. At slow dilution rates, we observed physiological changes that varied as a function of resource stoichiometry, including those linked to carbon and energy flow and storage. At high dilution rates cells aggregated, complicating chemostat dynamics, but in all treatments demonstrated similar biochemical and transcriptome responses despite differing resource supply ratios. Our study highlights the effects of elemental stoichiometry and growth rate on bacterial physiology and provides insight into the mechanisms required to sustain growth in the face of imbalanced resource ratios.

## MATERIALS AND METHODS

### Media composition

*Pseudomonas putida* KT2440 (DSM 6125) was grown in a modified version of COMBO medium originally developed for the growth of aquatic zooplankton and phytoplankton (32). Normal culture conditions included 200 mM C as glucose (33.3 mM), 10 mM N as NH_4_Cl, and 0.625 mM P as K_2_HPO_4_, yielding a C:N:P molar ratio of 320:16:1. We refer to this medium as “Balanced” throughout the manuscript as it (i) reflects an N:P of typical organismal biomass (11, 33–35) and (ii) has a C:P ratio near the boundary between C- and P- deficient conditions of some bacteria (36). The medium was buffered with 40 mM HEPES to a final pH of 7.4. The medium was prepared by first autoclaving CaCl_2_ and Fe:EDTA dissolved in high-purity water (18.2 mega-ohm); this was cooled and a concentrated preparation of all remaining components, which had been filter-sterilized through a 0.2 µm polyethersulfone filter, was aseptically amended (Table S1).

C:N:P ratios in COMBO medium were manipulated to impose resource stress by decreasing the concentration of one element from Balanced medium conditions. Based on results from batch culture experiments (Fig. S1), C:N:P molar ratios used for the chemostats were 320:16:1 (Balanced), 40:16:1 (C-stress), 320:6:1 (N-stress), and 320:16:0.2 (P-stress). These elemental ratios are consistent with those previously observed to force resource stress in aquatic bacteria beyond a typical biomass C:N:P of ~70:16:1 (11, 35).

### Growth conditions

KT2440 was grown in chemostats at 20°C with a culture volume of 75 mL (37, 38). Cultures were grown at two different dilution rates to represent slow and fast growth: 0.12 h^−1^ and 0.48 h^−1^. The maximum specific growth rate at 20°C under batch culture conditions exceeded 0.5 h^−1^ (Fig. S1), and empirical observations in chemostats showed that planktonic KT2440 washed out at dilution rates exceeding 0.6 h^−1^ but not at 0.48 h^−1^. Hence, a dilution rate of 0.48 h^−1^ approached the maximum growth rate for this organism at 20°C. Previously reported maximum growth rates for KT2440 average ~0.6 h^−1^ at 30°C (27, 39, 40).

To initiate all experiments, a cryo-preserved glycerol (20% vol/vol) stock of KT2440 was streaked onto an LB agar plate (BD Difco, Thermo Fisher Scientific, USA) and incubated at 20°C. A single colony was picked, inoculated in Balanced COMBO medium, and incubated overnight with shaking. One mL of stationary-phase culture was inoculated into each chemostat and incubated overnight in batch mode. Each resource stress treatment was performed in quadruplicate chemostats. After each chemostat became turbid, chemostats were switched from batch to continuous culture with the dilution rate controlled using a peristaltic pump (Watson-Marlow 205S, 16 channel). For the low dilution rate, the flow rate was immediately set to 0.12 h^−1^, while for the high dilution rate (0.48 h^−1^) the chemostat flow rates were increased stepwise over a 24-h period. Oxygen (O_2_) was controlled using aquarium pumps and, in fully turbid cultures, was sustained at 150 µM (equivalent to ~55% saturation). The chemostat pH ranged between 6.5 and 7. Chemostats were sampled after cells reached steady state (five residence times at the appropriate dilution rate (41)).

### Sampling constraints

To sample chemostats, the entire culture volume was removed and immediately processed for downstream analyses. Cell aggregation (flocculation) was observed in all treatments at dilution rates exceeding 0.3 h^−1^ (see Results and Discussion). Cells were observed to disaggregate approximately 1 h after being removed from the chemostat. Therefore, all assays described below were completed within 30 minutes of sampling to minimize physiological changes. We acknowledge that this sampling interval may bias our results and interpretation.

### Residual nutrient quantification

The residual concentrations of media N and P remaining in each chemostat were quantified. The inflow medium (~20 mL) was filtered through a rinsed, 0.45 µm pore size, mixed cellulose ester (MCE) filter. Prior to the disassembly of each chemostat for sampling, equal volumes of culture outflow (~5 mL) from each chemostat treatment were pooled (~20 mL) and filtered as above. Filtrate was stored at −20°C. Ammonium (NH₄^+^) and soluble reactive phosphorus (SRP) were quantified using an Astoria A2 segmented flow analyzer (Astoria-Pacific, OR, USA). The percent concentrations of residual NH₄^+^ and SRP were calculated by dividing the N or P remaining in the outflow by the initial concentrations in the media. Because the outflow from each chemostat treatment was pooled, values represent averages of four replicate chemostats per treatment.

### Biomass determinations: Dry weight, cell counts, cell volumes, and ATP

To estimate biomass dry weight, chemostat culture (10 mL) was filtered onto a pre-weighed 25 mm diameter 0.2 µm GTTP polycarbonate filter (MilliporeSigma). The filter was dried at 105°C overnight, stored in a desiccator, and reweighed. Cell abundances were estimated by epifluorescence microscopy of DAPI-stained bacteria. One mL of culture was fixed with a final concentration of 3% formaldehyde overnight at 4°C. Cells were filtered onto a 25 mm, 0.2 µm GTTP filter, and frozen at −20°C. DNA was stained using DAPI Vectashield (Vector Laboratories) and cells were visualized using an epifluorescence microscope (Olympus BX53) at 1,000× magnification. At least 10 fields of view or 200 cells were counted per sample. Cell volumes were estimated assuming the cell shape was a spherocylinder (42). Adenosine triphosphate (ATP) concentrations were determined *via* luminescence production using the BacTiter-Glo Microbial Cell Viability Assay (Promega, WI, USA). Briefly, 100 µL of culture was mixed with an equal volume of BacTiter-Glo reagent and incubated in the dark at room temperature for 5 min. Luminescence was quantified using a luminometer (GloMax 20/20, Promega). ATP concentrations were calculated based on a standard curve made from ATP disodium salt (Sigma-Aldrich, MA, USA).

### Cellular phosphorus

To measure cell phosphorus content, 3 mL of chemostat culture was filtered onto a pre-combusted, acid-washed, 25 mm, 0.7 µm glass fiber filter (Whatman). Filters were dried overnight at 105°C and stored in a desiccator. Filters were then placed in a scintillation vial and combusted at 500°C for 5 h. Samples were hydrolyzed with 10 mL of 0.15 M HCl at 60°C for 1 h. Finally, samples were treated with ammonium molybdate and potassium antimonyl tartrate, which, in the presence of ascorbic acid, allows for the quantification of phosphorus (43).

### Cellular carbon and nitrogen

Particulate C and N contents were determined from a set of chemostats run in parallel that were sacrificed specifically for these measurements to obtain sufficient biomass. Approximately 50 mL of culture was centrifuged at 4,300 × *g* for 10 min at 4°C. The cell pellet was washed with high-purity water (18.2 mega-ohm), centrifuged, and the supernatant discarded. This was repeated three times. The cell pellet was dried overnight at 105°C, stored in a desiccator, and weighed. The C and N contents were determined using an Exeter Analytical CE-440. C:N, N:P, and C:P ratios are reported as averages of the means with standard error uncertainty propagation.

### Concentrations of DNA and RNA

DNA and RNA were quantified fluorometrically as previously described (44, 45) using the Quant-iT Ribogreen RNA Reagent and Kit (Thermo Fisher Scientific). One mL of culture was centrifuged at 22,000 × *g* for 10 min at 4°C. The supernatant was discarded and the cell pellet was frozen at −80°C. The pellet was resuspended in 300 µL of extraction buffer (1% N-lauroylsarcosine in 1× TE buffer), sonicated on ice for 2 min, and incubated for 2 h with shaking at room temperature. Samples were diluted 1:6 with ice-cold Tris-EDTA buffer and incubated for 15 min with agitation. Samples (75 µL) were added to a 96-well black microplate, amended with 75 µL Ribogreen, and incubated for 5 min in the dark. Fluorescence was measured at 485/30 nm excitation and 528/20 emission on a microplate reader (FLx800 Bio-Tek). Samples were then amended with 10 µL of RNAse (Promega) to remove RNA, incubated in the dark for 25 min, and fluorescence was remeasured. RNA and DNA concentrations were calculated based on the difference in fluorescence against a set of RNA and DNA standards.

### Lipids

Lipid contents were determined using previously described methods (46–48). One mL of culture was centrifuged, freeze-dried, and stored at −80°C. The lyophilized culture was homogenized in a 2:1 chloroform:methanol mixture and extracted using the microsulfophosphovanillan method. Standards were prepared by dissolving cholesterol in 2:1 chloroform:methanol. Samples and standards were read on a spectrophotometer (Agilent Cary 60 UV-Vis) at 525 nm.

### Protein

Total protein quantification was performed using the Thermo Scientific Coomassie Plus Kit (Thermo Fisher Scientific). One mL of culture was pelleted at 22,000 × *g* for 5 min and frozen at −80°C. The pellet was homogenized in 400 µL 30% trichloroacetic acid, incubated at 4°C for 30 min, and then centrifuged at 15,500 × *g* at 4°C for 10 min. After the supernatant was removed, the pellet was rinsed with 5% TCA, treated with 300 µL of 0.2 M sodium hydroxide (NaOH), and vortex homogenized. Each sample (50 µL) was amended with 1.5 mL of Coomassie Plus Reagent (Thermo Fisher Scientific) and incubated for 10 min at room temperature. Protein content was determined colorimetrically at 595 nm on a spectrophotometer against protein standards ranging from 25 to 2000 µg mL^−1^.

### Oxygen consumption

Chemostat culture (15 mL) was diluted with 150 mL of the appropriate medium and immediately placed into a glass serum bottle equipped with an Oxygen Sensor Spot optode (PreSens). Optodes were affixed to the inside of the bottles and fiber optic cables were attached to the outside for light excitation and detection of emission. Bottles were crimp-sealed and O_2_ concentrations were measured in the dark over 20 min to estimate rates of consumption. Cultures were diluted into fresh media because respiration rates were faster than our ability to set up the experiment before O_2_ was completely consumed (e.g., 5 min). As our measurements were made following the dilution of cells into fresh media, they likely reflect an upper limit on rates of O_2_ consumption for each treatment.

### Transcriptomic sequencing

Culture (~15 mL) from each of four replicate chemostats was centrifuged at 4,300 × *g* for 10 min at 4°C and the supernatant was discarded. The cell pellet was submerged in RNAlater (Thermo Fisher Scientific) and stored at −20°C. Total RNA was extracted using the RNeasy Mini Kit (Qiagen, Germany). RNA library preparation, rRNA depletion, and sequencing on an Illumina Novaseq were performed using protocols recommended by the manufacturers (Novogene, Inc., Sacramento, CA).

Raw reads were cleaned with Trimmomatic v0.39 (49). Read recruitment against the KT2440 genome (NCBI accession GCA_000007565; 23, 50) was performed using Bowtie 2 v2.3.5.1 (51) and SAMtools v1.10 (52). Recruitment against each gene was quantified using featureCounts (53). Functional annotation was performed using NCBI Prokaryotic Genome Annotation Pipeline annotations (54) and GhostKOALA (55) against the KEGG database (56). Reads that mapped to ribosomal RNA genes were removed from further analysis. Gene expression was normalized using the metric Transcripts Per Million (TPM) for comparison and visualization. To show transcriptional differences of multiple treatments at the same time, we averaged the TPM of each gene across replicates within a given treatment and then normalized its expression relative to other treatments using the R package GGtern (57). We compared treatments using NMDS ordinations based on Bray-Curtis dissimilarities of rarefied recruitment counts with vegan (58). We tested whether resource stoichiometry and dilution rate were statistically significant drivers of transcriptome composition using *adonis* in vegan. DESeq2 (59) was used on unrarefied gene counts to identify differentially expressed genes between different resource stresses and dilution rates. To identify potential pathways that were differentially expressed, genes were grouped into functional categories based on KEGG pathway annotations using KEGGREST (60). Figures were made using ggplot2 (61) and BioRender (https://biorender.com/).

## RESULTS

### Elemental and biomolecular pools

We assessed the physiological response of *P. putida* KT2440 to differences in resource stoichiometry and chemostat dilution (growth) rate based on measurements of elemental and biochemical pools, cell morphology, O_2_ consumption, and transcriptome composition. Dry weight yield was greatest (0.5 g L^−1^) in the Balanced treatment and lowest in the P-stressed treatment (0.2 g L^−1^) at both dilution rates (Fig. S2). Cell abundance at 0.12 h^−1^ (slow dilution) was typically 3 × 10^8^ cells mL^−1^ under all conditions except P-stress, where cell densities were ~1 × 10^8^ cells mL^−1^. Dry weight cell^−1^ and volume cell^−1^ at 0.12 h^−1^ were lowest in the C-stressed treatment (Student’s *t*-test, C vs Balanced, *P* < 0.05), while ATP dry weight^−1^ was highest under C- and lowest under P-stress (C vs P, *P* < 1 × 10^−4^). However, ATP cell^−1^ was largely invariant across treatments at the slow dilution rate (averaging ~2 × 10^−9^ nmol cell^−1^; ANOVA, *P* < 0.61), consistent with ATP concentrations reflecting cytoplasm volume rather than biomass *per se* (62).

One of the most obvious phenotypic shifts that accompanied increases in dilution rate was cell autoaggregation and biofilm formation. At a dilution rate of 0.48 h^−1^ cells tended to clump, forming visually apparent aggregates containing hundreds of cells with combined lengths that could exceed 70 µm. This behavior was evident across all resource ratios and prevented accurate enumeration of cell abundances. Visual observation showed that cells released from aggregates after 1 h of removal from the chemostat. The tendency to aggregate and form biofilms complicates the assessment of growth rate; previous studies have found that cells within aggregates and biofilms can grow at different rates (63, 64), which in chemostats may violate the assumption of steady-state behavior. The increase in dilution rate clearly triggered a phenotypic response (cell aggregation), but it remains unclear the extent to which the cells in these high dilution treatments were uniformly growing at the specified growth rate (0.48 h^−1^) or whether the aggregation response resulted in a mixed population of fast and slow growing cells. Given this uncertainty, we refer to the dilution rate rather than the growth rate when comparing treatments.

**Fig 1:**
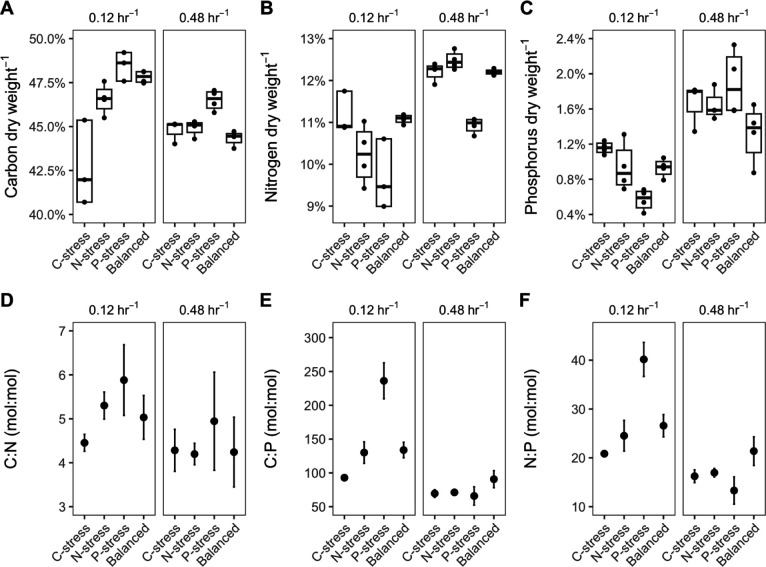
*P. putida* KT2440 elemental stoichiometry varies with resource stoichiometry and dilution rate. Carbon (**C**), nitrogen (**N**), and phosphorus (**P**) per dry weight (**A, B, C**) and their molar ratios (**D, E, F**). Note that Y-axes are not the same across plots.

For each treatment, we measured cellular C, N, and P contents (Fig. 1). Together, these three elements accounted for ~50%–60% of the observed KT2440 dry weight. At the slow dilution rate, carbon accounted for a smaller proportion of the dry weight when cells were grown under C-stressed conditions, while cells became increasingly carbon-rich and larger under P-stressed conditions, representing ~43% and 48% of the dry weight, respectively (C vs. P, *P* < 0.042). By contrast, dry weight-normalized N contents were lowest under P- and N-stress and greatest under C-stress, ranging between ~9.5% and 12.5% (C vs. P, *P* < 0.07). P accounted for a significantly lower fraction (~0.6%) of the dry weight under P-stress than the other treatments at the slow dilution rate. As a result, C:N, C:P, and N:P molar ratios were highest under P- (~5.9, 236, 40, respectively) and lowest under C- (~4.5, 93, 21) stress. Elemental contents and ratios in the Balanced medium treatment, which was intended to represent optimal resource ratios for growth, generally fell between values observed in the N- and P-stressed treatments. At the faster dilution rate, C, N, and P contents per unit dry weight and the resulting molar ratios were more similar among treatments: for example, C, N, and P contents in all treatments averaged 45%, 12%, and 1.6%, respectively, while the C:N, C:P, and N:P ratios averaged 4.2, 70, and 15, indicating average biomass C:N:P of ~70:15:1. Coefficient of variation of resource ratios within all treatments decreased with increasing dilution rate (C:N, 0.11 to 0.07; C:P, 0.41 to 0.24; N:P, 0.32 to 0.27).

We also measured cellular pools of protein, lipid, RNA, DNA, and ATP (Fig. S3). Together, these macromolecular pools represented ~45%–60% of the dry weight. At the slow dilution rate, protein represented 14%–25% of the dry weight, with P-stressed cells demonstrating the lowest protein content, while C-stressed treatments demonstrated elevated protein content. Protein content increased (23%–30% of dry weight) with dilution rate across all treatments (*t*-test, *P* < 1 × 10^−5^). Total lipids exhibited opposite patterns. At slow dilution rates, the lipid content of C-stressed cells was lowest (14%), while P-stressed cells were enriched in lipids (25% dry weight; C vs P, *t*-test, *P* < 1 × 10^−3^). Across all resource treatments, lipid content decreased at faster dilution rates (0.12 h^−1^ vs 0.48 h^−1^, *t*-test, *P* < 0.02). RNA content represented ~10% of the biomass and increased with dilution rate in all treatments except Balanced conditions (*t*-tests, *P* < 0.05). Cells under P-stress showed the most dramatic changes in RNA with dilution rate, increasing from 3% of the dry weight at 0.12 h^−1^ to 10% at 0.48 h^−1^. DNA and ATP represented small proportions of the dry weight under all conditions, comprising <3% and <0.13%, respectively. Similar to elemental content and stoichiometry, macromolecular content per dry weight converged at a higher dilution rate regardless of media resource composition.

### Slow-growing cells adjust their physiology to acquire the limiting resource

**Fig 2:**
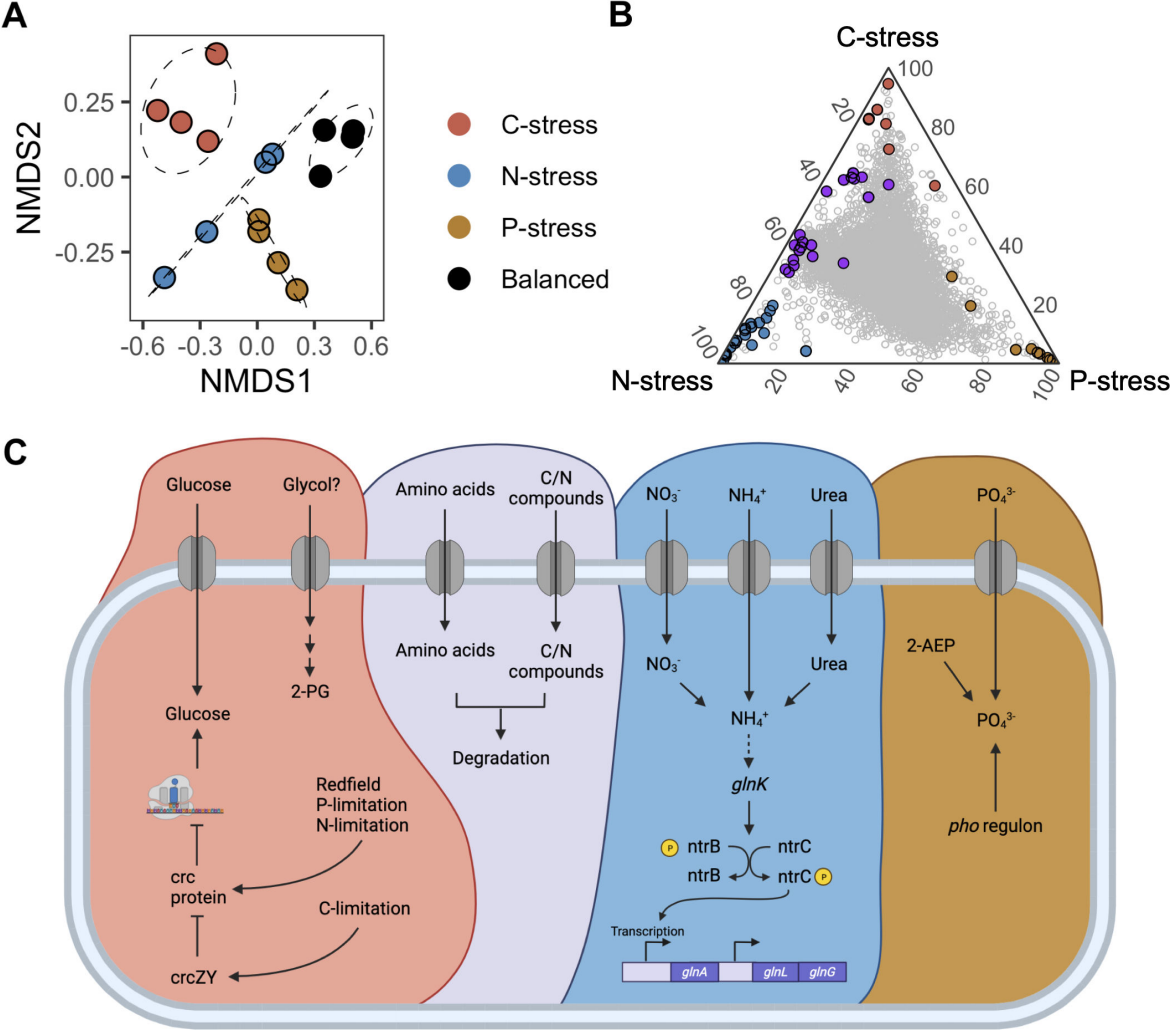
The major transcriptional response to variable resource stoichiometry at a dilution rate of 0.12 h^−1^ (slow growth) is the expression of genes to obtain the limiting resource and their global regulators. (**A**) Non-metric multidimensional scaling (NMDS) ordination based on Bray-Curtis dissimilatory of transcriptomes. Circles represent the 75% confidence interval for each resource ratio. (**B**) Ternary plot comparing average relative percent transcript expression between C-, N-, and P-stressed resource treatments, where each gray circle represents a gene. Genes of interest are shown in color. (**C**) Cartoon highlighting major pathways involved in nutrient uptake and global regulation within each treatment. The colors in A, B, and C are the same; for example, the genes enriched under C-stress are red in all panels. Genes in purple in B and C are shared under C- and N- stress.

We used transcriptomic sequencing for mechanistic insights into the adaptive strategies of KT2440 in response to variations in resource stoichiometry and dilution rate. When comparing transcriptomes of cells cultured at 0.12 h^−1^, the type of resource stress was a statistically significant predictor of transcriptome composition (PERMANOVA, R^2^ = 0.747, *P* < 0.001). There were 2,576, 1,139, and 1,847 differentially expressed genes when comparing C- and P-, C- and N-, and N- and P- stressed treatments, respectively (Tables S2 to S8), indicating that, at a fixed dilution rate, different kinds of resource stress induce distinct patterns of transcription. The most differentially expressed genes among treatments were involved in the uptake and assimilation of whichever resource was limiting, including glucose, ammonium, and phosphate (Fig. 2). These transcripts were likely controlled by increased expression of key regulatory pathways. For example, under C-stress, KT2440 expressed the non-protein-coding RNAs *crcZY* to sequester the Crc protein, thereby inhibiting carbon catabolite repression and releasing genes for glucose assimilation (65–67). Under N- and P-stress, respectively, the *gln* and *pho* regulons were highly expressed, operons that function in sensing and responding to N and P deficiency (26, 68). Interestingly, KT2440 also expressed transcripts that encode proteins for the uptake of C, N, and P in forms that were not provided in the media. For example, C-stressed cells transcriptionally expressed a putative porin involved in the uptake of ethylene glycol (69), N-stressed cells expressed genes for the transport of nitrate and urea and their conversion to ammonium before assimilation, while P-stressed cells transcribed *phnXW* whose products function in the degradation of 2-aminoethylphosphonate. Furthermore, cells in some treatments shared expression of transcripts that were largely absent in the remaining treatment. For example, under C- and N-stress, transporters involved in the uptake and degradation of compounds that had both C and N moieties, such as amino acids, were expressed. By contrast, transcripts for the synthesis and turnover of RNA and proteins were elevated in the N- and P-stressed treatments relative to C-stress, including elongation factor P, an endonuclease, a polyribonucleotide nucleotidyltransferase, and a putative protease (26). Relatively, fewer transcripts were shared by C- and P-stressed cells.

### Central carbon metabolism and energy flux

Given differences in biomass C content between treatments, we assessed pathways involved in central C metabolism. Transcripts involved in glucose catabolism and the tricarboxylic acid (TCA) cycle (70) were more highly expressed under P- and N- relative to C-stress (Fig. S4). Transcripts involved in fatty acid biosynthesis and degradation, along with genes that function in the synthesis, structure, and degradation of polyhydroxyalkanoates (PHAs) were especially enriched under P-stress. The general trend toward higher relative expression of these pathways under P-stress was maintained even at fast dilution rates, consistent with elevated C:P ratios and lipid contents of these cells.

**Fig 3:**
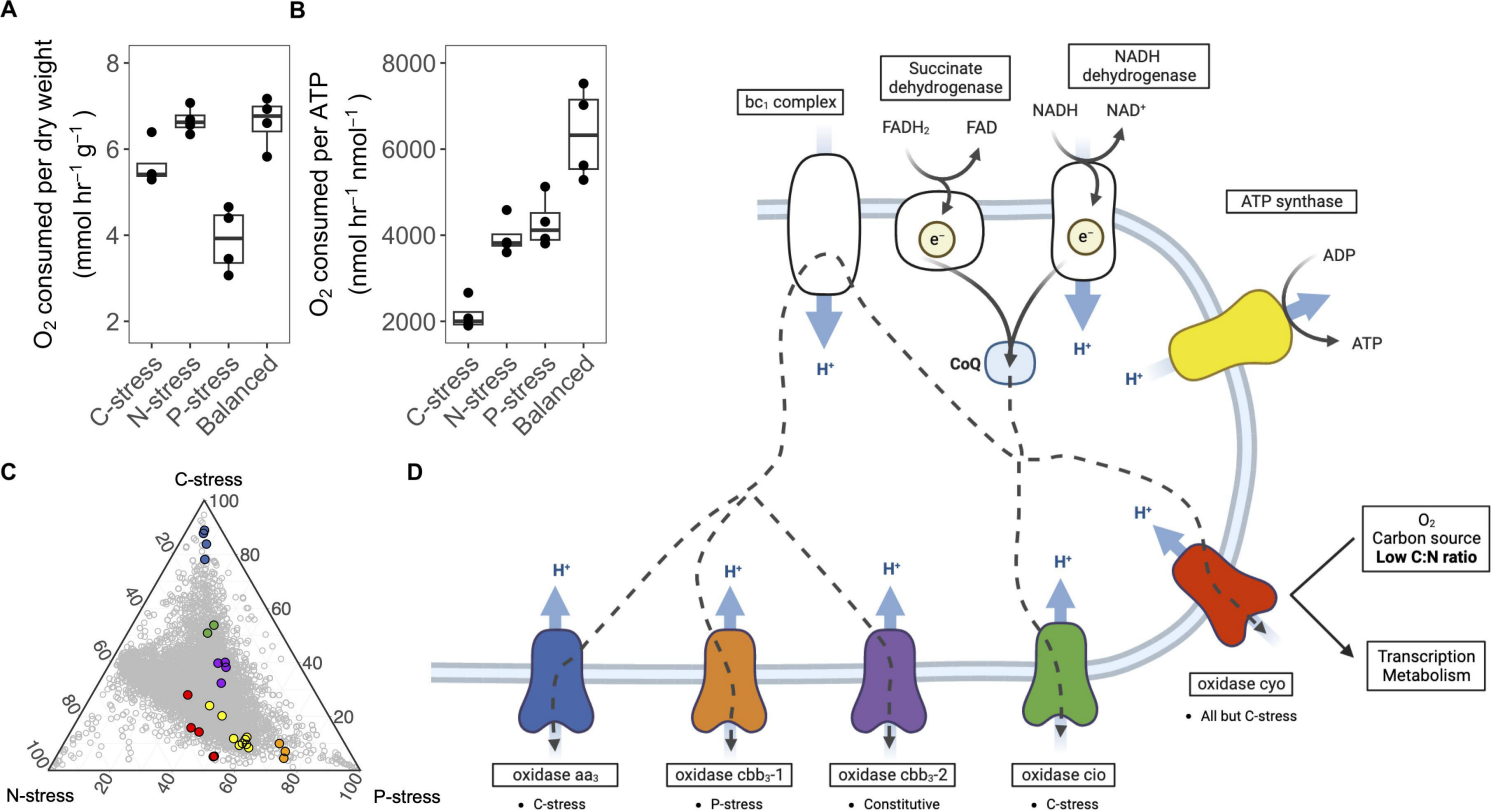
Respiratory changes appear critical to adaptation to variable resource stoichiometry at a dilution rate of 0.12 h^−1^. (A and B) Oxygen consumption per dry weight and per ATP. Note that Y-axes are not the same between plots. (**C**) Ternary plot comparing relative percent transcript expression between C-, N-, and P-limited resource ratios at 0.12 h^−1^, where each gray circle represents a gene. Respiratory genes of interest are represented in color. (**D**) A cartoon of the electron transport chain of *P. putida* KT2440. The colors of the terminal oxidases and ATP synthase in panel D reflect the genes in C.

Differences in C metabolism would be expected to have ramifications for energy generation, respiratory O_2_ consumption, and growth efficiency. When normalized to dry weight, rates of O_2_ consumption were lowest in slow dilution rate P-stressed cells (C vs P, *P* < 0.01); however, when normalized to ATP (or per cell), C-stressed cells demonstrated the lowest rates of respiration (Fig. 3; C vs P, *P* < 1.7 × 10^−3^). Here again, we leveraged the transcriptome for potential clues into the underlying cause of these metabolic differences. The five terminal oxidases involved in the electron transport chain showed resource-stress-specific differences in expression at slow growth (Fig. S5). C-stressed cells expressed the *aa_3_* oxidase and to a smaller extent *cio*, while the *cbb_3_-1* oxidase was more highly expressed under P-stress. Transcripts encoding proteins that catalyze the first step of electron transfer and final step of ATP generation, such as *nuo* dehydrogenase, succinate dehydrogenase, and ATP synthase, were less abundant under C-stress. These observations indicate that C-stress alters energy-generating pathways.

### Dilution-rate-dependent responses

**Fig 4:**
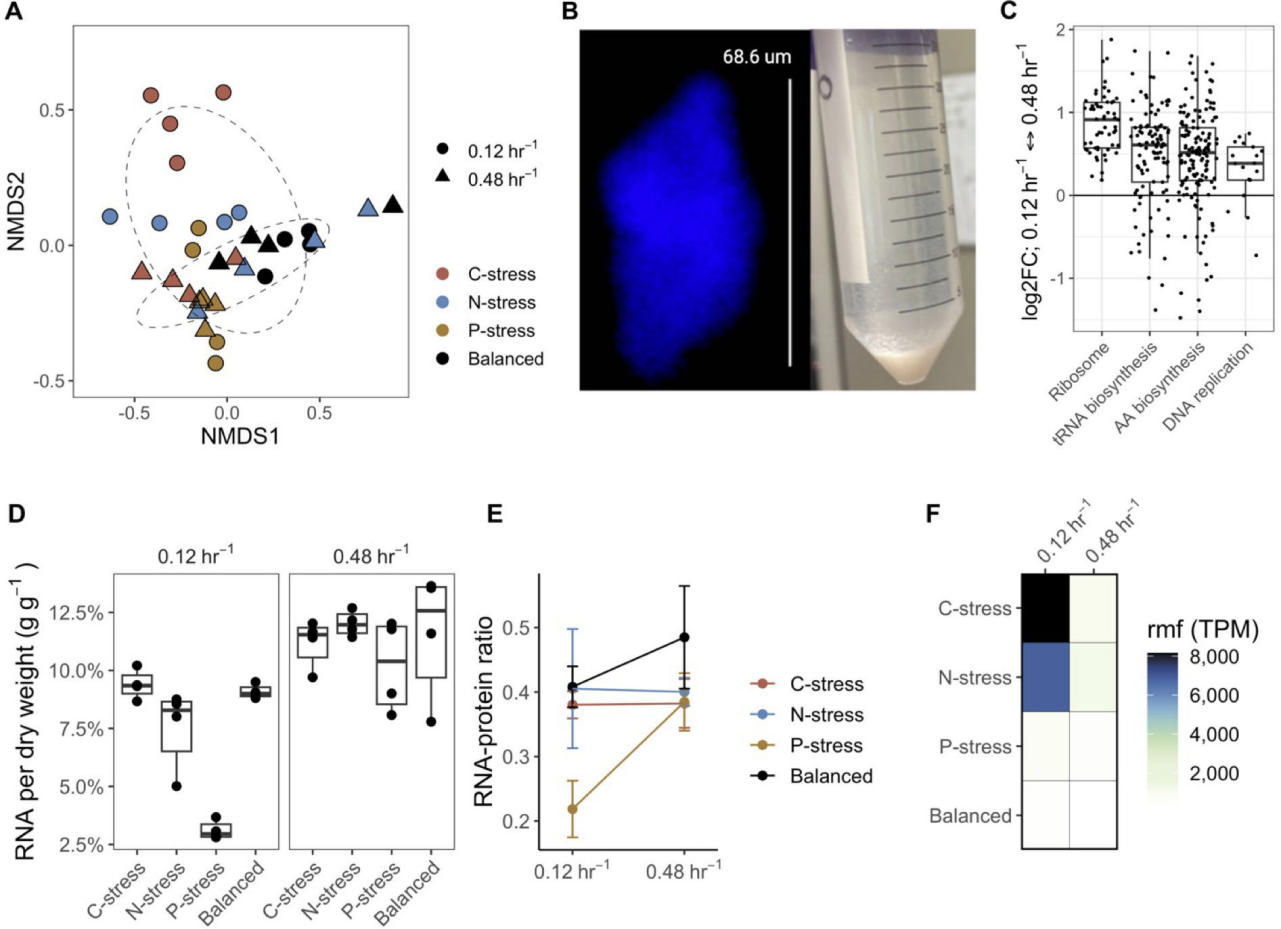
*P. putida* KT2440 physiology converges at fast dilution rates despite differences in resource stoichiometry, with specific changes in RNA. (**A**) Non-metric multidimensional scaling (NMDS) ordination based on Bray-Curtis dissimilatory of transcriptomes at different dilution rates and resource treatments. Circles represent the 75% confidence interval of each dilution rate. (**B**) A fluorescence microscopy image (with white scale bar) of a cell aggregate and photograph following culture removal from the chemostat showing flocculation at 0.48 h^−1^. (**C**) Expression of genes involved in the pathways of ribosome synthesis, tRNAs, amino acids, and DNA replication as a function of dilution rate. Log2FC; log2FoldChange. (**D**) RNA content per dry weight. (**E**) The ratio of total RNA to total protein at different dilution rates and resource treatments. (**F**) Transcript expression (in transcripts per million, TPM) of the ribosome modulation factor (*rmf*).

NMDS ordination analysis based on Bray-Curtis dissimilarity showed that transcriptome composition across all treatments was less variable at a dilution rate of 0.48 h^−1^ relative to 0.12 h^−1^ (Fig. 4). Resource stoichiometry was a weaker predictor of transcript composition at the higher dilution rate relative to the slow dilution rate (R^2^ = 0.473, *P* < 0.024). When comparing all treatments together, both dilution rate and resource stoichiometry were statistically significant drivers of transcriptome composition (dilution rate, R^2^ = 0.11, *P* < 0.002; resource stoichiometry, R^2^ = 0.38, *P* < 0.001). Over 2,000 genes were differentially expressed between the slow and fast dilution rate treatments (Table S9). Many important metabolic genes that were differentially expressed at slow dilution rates, including those involved in cellular respiration, glucose catabolism, and the TCA cycle, did not differ as a function of resource stress at the faster dilution rate (Fig. S6). Nevertheless, even at fast dilution rates, cells in the P- and N-stressed treatments overexpressed transcripts for proteins involved in the transport and assimilation of P and N, respectively.

When comparing transcriptomes at fast versus slow dilution rates, some of the most differentially abundant genes included those associated with ribosomes. When grouping genes based on KEGG pathways, those involved in the synthesis of ribosomal proteins, tRNA biosynthesis, amino acid biosynthesis, and DNA replication were more highly expressed in cells at fast than at slow growth (Fig. 4). These changes were especially evident under P-stress and more modest for C- and N-stress, largely consistent with treatment-specific differences in biomass P and RNA content. For example, under N-stress, no ribosomal protein genes were differentially expressed at the faster dilution rate relative to the slower one (Fig. S7). The RNA to protein ratio, which is known to increase with growth rate (71–74), increased with dilution rate under P-stress and balanced conditions but not in C- or N-stressed cells. Departures from the expected increase in the RNA:protein ratio with growth rate could reflect the storage of ribosomes under slow growth conditions. Consistent with this idea, the ribosome modulation factor (*rmf*), which stores ribosomes by dimerization (75), was highly expressed under C- and N-stress under slow dilution rates.

We searched for genes that could provide a mechanistic underpinning for cell autoaggregation at high dilution rates. KT2440 can aggregate and form biofilms *via* the synthesis and export of a variety of polymers, including alginate, cellulose, exopolysaccharides, and lap proteins, many of which are controlled post-transcriptionally (76–80). In our experiments, genes involved in these pathways were not enriched in the faster dilution treatments; rather, many were more highly expressed in the slower dilution rate treatments or were specific to only one resource stress condition (Table S2). For example, the response regulator *cfcR* (PP_4959) and its post-transcriptional regulator *rsmE*, which control the level of c-di-GMP and modulate biofilm formation (81–83), were both more highly expressed at 0.12 h^−1^. However, other genes involved in cell membrane biogenesis and flagellar synthesis were differentially expressed depending on the dilution rate. For example, *cfa* transcripts, responsible for the synthesis of cyclopropane fatty acids (CFAs), were enriched at slow dilution rates. Similarly, some gene clusters involved in flagellar synthesis, which are organized into at least 10 transcriptional units (84), were also more highly expressed at 0.12 h^−1^. Altogether, mechanisms underlying the phenotypic transition from free-living to aggregation that accompanied increased dilution rate were not readily apparent from the transcriptional analyses. These results are consistent with previous studies working with *P. aeruginosa* that indicate that mechanisms controlling cell aggregation are not universal within a single strain and can be difficult to identify due to specific environmental conditions (85).

### On balanced resource conditions

**Fig 5:**
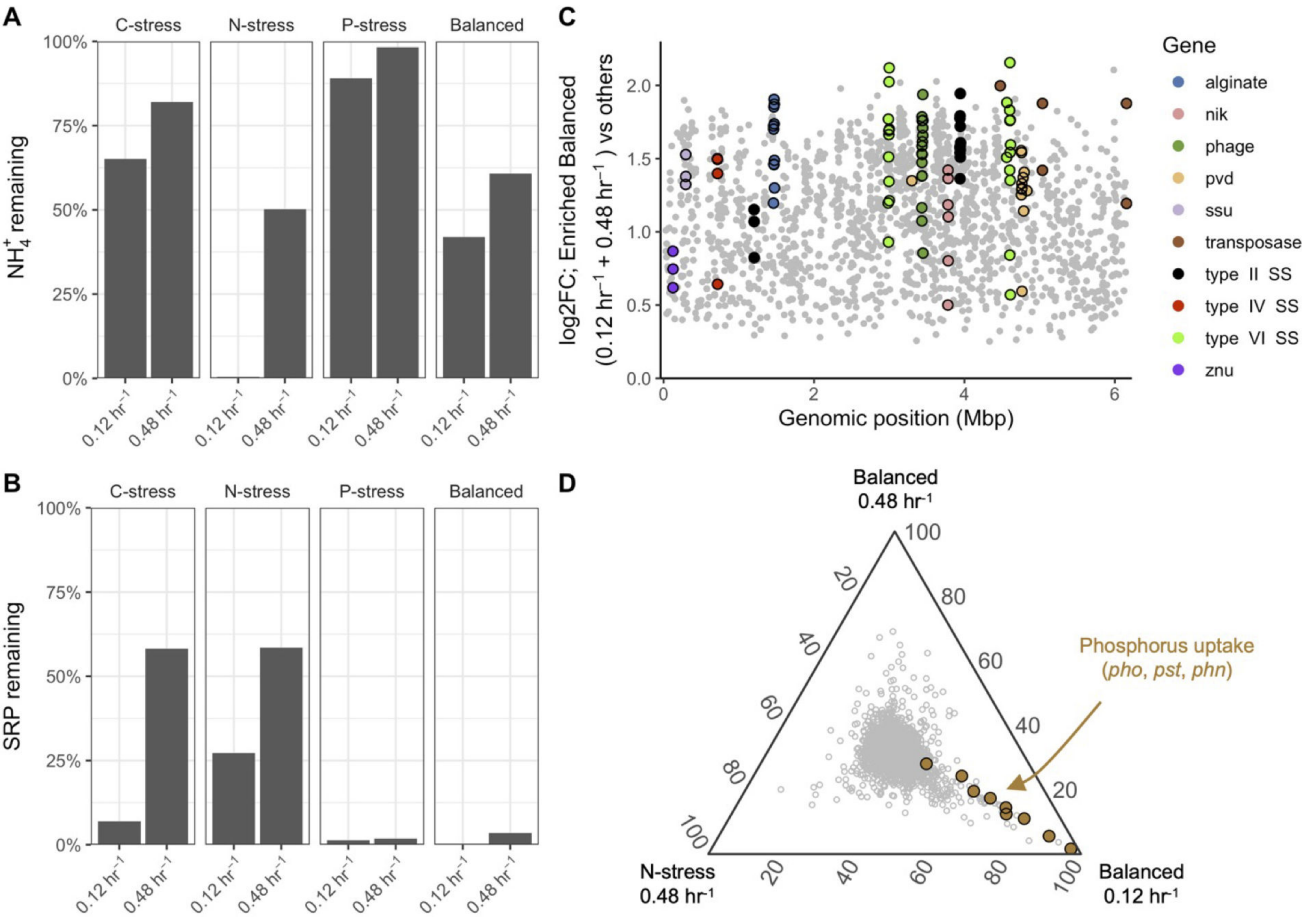
Cells supplied with Balanced medium C:N:P ratios appear phosphorus limited and express genes for the uptake of trace elements. (A and B) The proportion of the ammonium (NH_4_^+^) and soluble reactive phosphorus (SRP) remaining in the outflow as a function of nutrient stress and dilution rate. (**C**) Genes enriched (*P* < 0.05) under Balanced growth (0.12 h^−1^ and 0.48 h^−1^) when compared against all other treatments. Genes of interest are shown in color and labeled. (**D**) Ternary plot comparing average relative percent transcript expression between Balanced (0.12 h^−1^ and 0.48 h^−1^) and N-stress (0.48 h^−1^) resource ratios, showing that transcriptomes under N-stress at high dilution rates are similar to those in cells grown under Balanced resource ratios. Each gray circle represents a gene.

Finally, we compared the balanced medium condition (intended to represent optimal resource supply) against the various treatments designed to impose resource stress. Relative to the C, N, or P- stressed treatments, we found that cells in the balanced medium were enriched in transcripts for transposases, phage-associated genes, secretion systems, and the production of alginate, genes which were not highly expressed overall but were nonetheless differentially abundant (Fig. 5; Table S10). Cells grown in the balanced treatments also had higher expression of transcripts for the uptake of micronutrients, including sulfonates (*ssu*), zinc (*znu*), nickel (*nik*), and the pyoverdine (*pvd*) operon that encodes for the production of siderophores and iron uptake. Taken together, these observations suggest that under optimal conditions, when cells are not as limited by C, N, and P, they may become limited by trace elements used as protein co-factors. Interestingly, many of these genes were also expressed by N-stressed cells at high dilution rates but not under C- and P-stress, highlighting similarities among cells under balanced growth at both dilution rates and N-stressed cells at high dilution rates (Fig. 5; Fig. S6; Table S11). Such findings are consistent with analyses of residual nutrients in the chemostats: at high dilution rates, both N-stressed and balanced chemostats had residual N and P, suggesting some other nutrient limited complete consumption of these elements in the chemostats.

## DISCUSSION

### Cells activate multiple pathways that transport and assimilate compounds containing the growth-limiting element

Here we evaluated the response of *Pseudomonas putida* KT2440 to changes in C, N, and P stoichiometry and dilution (growth) rate. Treatment-specific patterns of elemental stoichiometry and transcriptome expression were evident at slow dilution rates of 0.12 h^−1^. One of the strongest responses to resource stress was the expression of regulatory pathways and transcripts related to the acquisition and assimilation of the growth-limiting element. This finding is consistent with previous work showing pseudomonads have a robust regulatory network that enables them to control their response to resource limitation (86–88). Under C-stress, KT2440 expressed *crcZY*, non-coding RNAs that sequester the Crc protein and release carbon catabolite repression of glucose assimilation (65–67, 89). Under N-stress, the *gln* operon, which regulates dozens of genes involved in N and C metabolism (26), was highly expressed. Our findings provide further support that the Crc and Ntr regulatory systems help regulate C:N biomass stoichiometry in *Pseudomonas* (26, 65, 67, 86, 87, 90, 91), especially under conditions of slow growth. We note that KT2440 also expressed transcripts for the acquisition of C, N, and P sources that were not supplied in the media. In our experiments, cells responded to C-stress by seeking to acquire not only glucose but also glycolate and glyoxylate. N-stressed cells activated pathways to acquire the ammonium supplied in the medium but also alternative forms of nitrogen, including nitrate, urea, and amino acids. Similarly, cells under P-stress expressed genes for the incorporation of not only phosphate but also phosphonates. Finally, both C- and N-stressed cells expressed transcripts for the uptake of alternative compounds containing both C and N moieties, such as amino acids and compatible solutes. Altogether, these observations show that KT2440 activates multiple pathways to acquire essential elements that are limiting to growth regardless of their form, including by recycling intracellular content, accessing metabolic byproducts, and scavenging dead extracellular material as cells decompose (7, 92, 93).

### Different forms of resource stress induce specific changes in how carbon is partitioned between catabolic and anabolic pathways

Our findings also highlight how resource stress can induce changes in energy generation and carbon storage in slow-growing cells. For example, P stress resulted in increases in cellular C reserves, C:N and C:P ratios, and total lipids, coincident with elevated transcripts related to C metabolism and storage. By contrast, C-stress cells had lower cellular C and C:N and C:P ratios. These observations may be in part due to C storage as polyhydroxyalkanoates in P- and N-stressed cells. PHAs act as carbon and energy reservoirs to minimize energy spillage and may constitute up to 80% of cell dry weight under nutrient-limited conditions (94–101). Furthermore, our findings suggest that C-stressed cells maintained lower rates of respiration per ATP (or per cell), while P-stressed cells appeared to use less oxygen per unit dry weight despite greater rates of respiration per cell. We see evidence that underlying these changes in C storage and growth efficiency were modifications in respiratory electron transport pathways. KT2440 contains at least five terminal oxidases, each of which exhibits a unique redox potential, affinity for O_2_, and ability to pump protons for ATP generation. These alternative pathways allow KT2440 to regulate respiration based on O_2_ concentration, C source, growth phase, and nutrient limitation (98, 102–104). The *cyo* terminal oxidase, which was preferred at fast dilution rates in our experiments, acts as part of a global regulatory network that senses electron transport chain activity and influences the expression of hundreds of genes (102, 103, 105). At slow dilution rates, C-stressed cells used an aa_3_-type oxidase preferentially to cyo, while P-stressed cells appear to rely more heavily on the cbb_3_-1 oxidase. Oxidases of the aa_3_-type can have higher proton-translocating efficiency than their cbb_3_ counterparts (106–108). Hence, the aa_3_ oxidase may function as an energy conservation mechanism under low C conditions (109). Changing the terminal oxidase from cyo to aa_3_ may have dual purposes for C-stressed cells: it could alter the expression of genes otherwise controlled by cyo and also optimize ATP generation in the face of decreased reductant flux and energy deficiency. Because we measured ATP pools, not fluxes, a valuable direction for future work would be to estimate ATP turnover under different types of resource stress (110, 111).

### Cell auto-aggregation is a generic response to high dilution rate, regardless of resource limitation

One of the most visible phenomena we observed was autoaggregation of cells when grown at fast dilution rates. This switch in phenotype complicates the assessment of growth rate because flocculation could decouple dilution rate from cellular growth: aggregated and biofilm-associated cells have previously been shown to vary in growth rate, elemental ratios, and gene expression (104, 112, 113). The reasons for autoaggregation and biofilm formation can vary but can reflect a physiological mechanism that allows cells to persist in nutrient-enriched locations or to minimize cell stress (114, 115). Consistent with our study, previous work has shown that aggregative behavior by strains of *P. putida* occurs at high dilution rates (116–119). Similar aggregative behavior has also been reported in *Enterococcus faecalis*, *Escherichia coli*, *Staphylococcus aereus*, and *P. aeruginosa* during exponential growth (120, 121), with the latter growing predominantly as aggregates during conditions of fast growth (85, 122). Both *P. putida* and *P. aeruginosa* disperse from biofilms in response to starvation (29, 30, 122). Consistent with this observation, we found that at slow dilution rates, KT2440 differentially expressed transcripts for the production of cyclopropane fatty acids, ring-containing lipids synthesized in response to adverse conditions and during entry into the stationary phase (123–127), as well as flagellar transcripts that would be required for dispersal. Therefore, we hypothesize that KT2440 forms aggregates when growing rapidly under favorable conditions and disaggregates when resources become scarce. This physiological behavior may be both common (128) and important as bacteria approach μ_max_, allowing cells to remain in a fixed, nutrient-rich location by preparing for surface attachment and biofilm formation. Further work will be needed to clarify the significance and mechanisms of this behavior in KT2440. This would require using near-instantaneous sampling protocols as aggregation responses can be transient.

### *P. putida* cultured at high dilution rates converges on a common physiological phenotype regardless of media composition

Although cell aggregation complicates the use of dilution rate as a measure of growth rate in chemostats, we observed physiological changes that suggest KT2440 was growing faster at 0.48 h^−1^ than at 0.12 h^−1^. For example, while biomass elemental stoichiometry and transcriptome expression were flexible at the slow dilution rate, they were less variable and converged across treatments when the dilution rate approached μ_max_. These findings are consistent with the observation that elemental stoichiometry of bacterial biomass varies depending on resource conditions, especially at slow growth, but converges at fast growth rates near a C:N:P of ~70:15:1 (9, 14). Similar trends have been reported for transcriptome composition, which shows a dependence on growth rate (129, 130) and convergence at fast growth (131). We also found that RNA became a larger proportion of cell biomass at faster dilution rates, consistent with previous studies that have documented positive relationships between cellular RNA pools and growth rate (71, 132–136). However, in our experiments, the strength of the RNA-growth rate relationship varied depending on resource stoichiometry. For example, increases in cellular RNA were most pronounced under P-stress, but more modest under C- or N-stress, in agreement with a previous study that found no change in RNA content as a function of growth rate under C-limitation in KT2440 (27). Bacterial P content can be highly flexible (137), suggesting differences in the fraction of active ribosomes and protein elongation rates may account for differences in RNA content (73, 138, 139). One mechanism for altering the number of active ribosomes is ribosomal hibernation *via rmf* (140), a gene that was expressed by KT2440 here under C- and N-stress at slow growth. Elevated expression of *rmf* has been observed in C- and N-limited *E. coli* (74), and *rmf* mutants show an inability to control ribosome abundance (141). The elevated pools of RNA that we observed at slow growth under C- and N- stress suggest that KT2440 maintains excess, stored ribosomes when P resources are abundant, permitting rapid increases in protein synthesis when limiting resources once again become available (5, 73, 135). Altogether, our data point to physiological changes that would be consistent with increases in growth rate as a function of dilution rate and that cell physiology is less variable as cells approach μ_max_ even under different resource ratios.

### Changes in luxury gene expression may be one way cells respond to resource conditions

Finally, we explored the response of cells to more optimal resource conditions (Balanced medium treatment) where the N:P supply ratio matched the commonly observed cell biomass ratio of 16:1 (11, 33–35). Phenotypic and residual element concentrations indicated that cells in the Balanced medium behaved somewhat differently from the other treatments, suggesting a distinct physiology intermediate to N- or P-stress. Interestingly, we observed similar transcriptomic patterns in the Balanced treatments at both dilution rates and under N-stress at the fast dilution rate (0.48 h^−1^). These findings were consistent with analyses of residual elements in the chemostats: at fast growth, both N-stressed and Balanced treatment chemostats had appreciable residual N and P. One explanation could be that as growth rates approach μ_max_, residual concentrations of the limiting resource increase (142). Nevertheless, transcriptomic data may be consistent with the interpretation of a shift toward an alternative limiting resource at high growth rates. For example, shared transcripts specific to these three treatments (Balanced at 0.12 h^−1^ and 0.48 h^−1^, N stressed 0.48 h^−1^) included those for the uptake of micronutrients, including the pyoverdine operon for the production of siderophores (pvd), sulfonates (ssu), zinc (znu), and nickel (nik), consistent with limitation by trace elements. We hypothesize that a dilution rate of 0.48 h^−1^ may have relaxed N-stress but increased requirements for micronutrients such as sulfur, iron, or zinc. Future work should consider the role of growth rate on cellular micronutrient requirements (143). Interestingly, these three treatments also expressed luxury transcripts not required for growth in monoculture but which may provide an advantage in natural settings when competing with other organisms. Resource limitation can lead to downregulation of genes that potentially carry a fitness cost, including transposases (144), secretion systems (145), siderophores (146), and virulence traits (147, 148). We hypothesize that under fast growth when C, N, or P may be less limiting, luxury genes are expressed; however, downregulation of these genes, which are unnecessary to maintain growth and may carry a metabolic burden, could be one mechanism to deal with resource stress.

## Data Availability

Transcriptomic data are publicly available under NCBI accession PRJNA1063263.

## SUPPLEMENTAL MATERIAL

Supplemental Figures msystems.00770-24-s0001.docx Figures S1 to S7.

Supplemental Tables msystems.00770-24-s0002.xlsx Tables S1 to S11.

ASM does not own the copyrights to Supplemental Material that may be linked to, or accessed through, an article. The authors have granted ASM a non-exclusive, world-wide license to publish the Supplemental Material files. Please contact the corresponding author directly for reuse.
