# Supplementary material for: Physiology, fast and slow: bacterial response to variable resource stoichiometry and dilution rate
Source: mSystems. 2024 Jul 9;9(8):e00770-24. doi: 10.1128/msystems.00770-24 (PMC11334502; doi:10.1128/msystems.00770-24)
Supplement: Supplemental Figures — Figures S1 to S7. [file msystems.00770-24-s0001.docx]

**Supplemental Figure 1**. Batch culture growth curves of *P. putida* KT2440 when grown under different resource ratios. A C:N:P ratio of 320:16:1 reflects concentrations as described for the Balanced treatment. Nutrient stress was forced by decreasing the concentration of one nutrient, including modifications in the concentrations of carbon (**A**), nitrogen (**B**), and phosphorus (**C**). **D**) The area of maximum slope (growth rate; 0.5 hr^-1^) of all treatments combined.


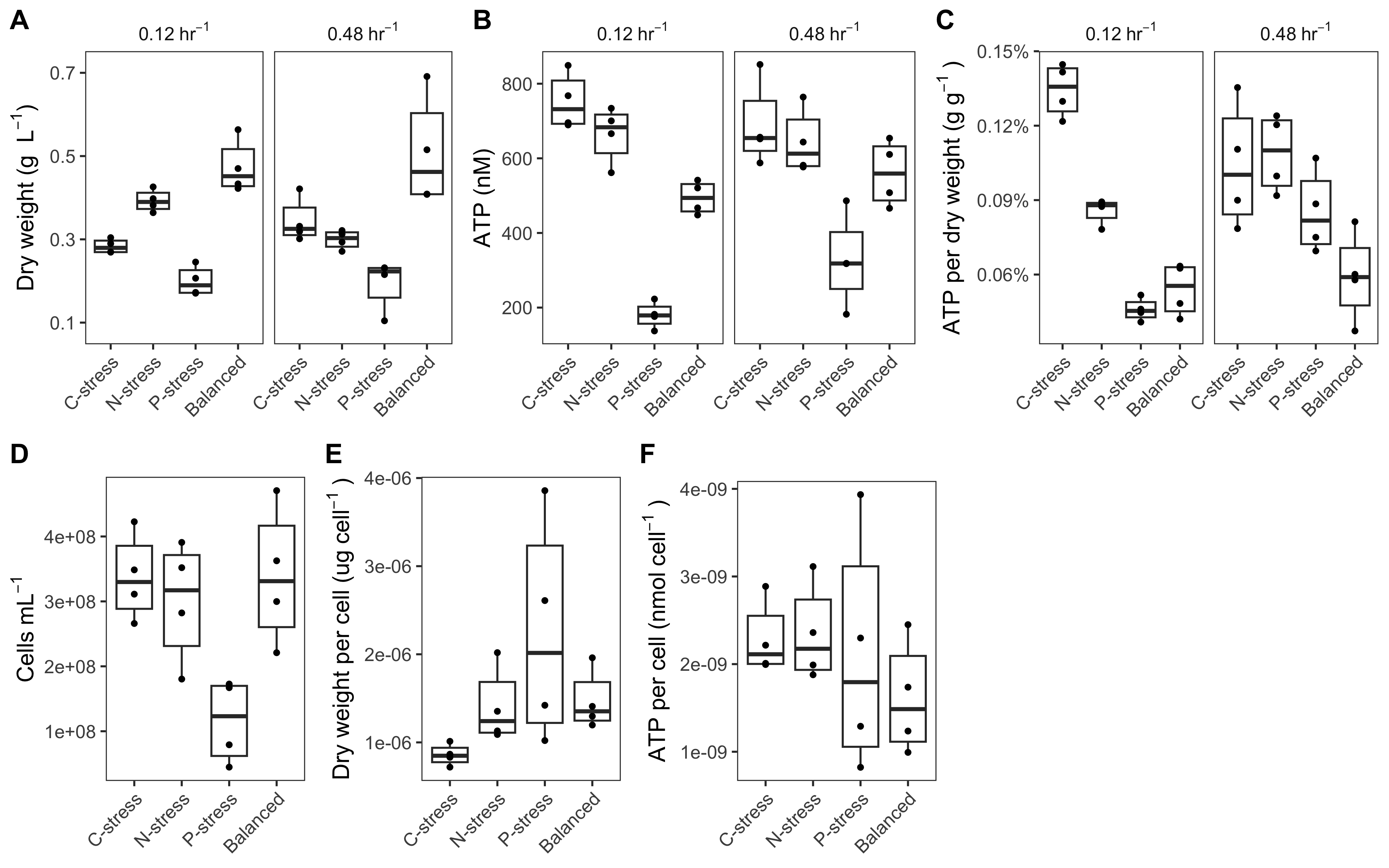


**Supplemental Figure 2**. Biomass measurements as a function of resource stoichiometry and growth rate. Biomass estimates include dry weight (**A**), ATP (**B**), and ATP per dry weight (**C**). Cell counts (**D**), dry weight per cell (**E**), and ATP per cell (**F**) were determined at 0.12 hr^-1^. Note that Y axes are different across plots.

**Supplemental Figure 3**. Biochemical pools per dry weight shown as a function of dilution rate and resource stoichiometry.

**Supplemental Figure 4**. C- and P- stress induces changes in carbon metabolism and storage. **A**) Ternary plot comparing average relative percent transcript expression between C-, N-, and P- stress resource stoichiometry at 0.12 hr^-1^, where each grey circle represents a gene. Genes involved in the TCA cycle (blue) and the EDEMP pathway (orange; consisting of genes belonging to the Entner-Doudoroff, Embden-Meyerhoff-Parnas, and pentose phosphate pathway) are colored. **B**) Lipids per dry weight across resource ratios and growth rates. **C**) Transcript expression of genes involved in fatty acid biosynthesis (top), polyhydroxyalkanoate synthesis and degradation (middle), and fatty acid β-oxidation (bottom). TPM, transcripts per million.


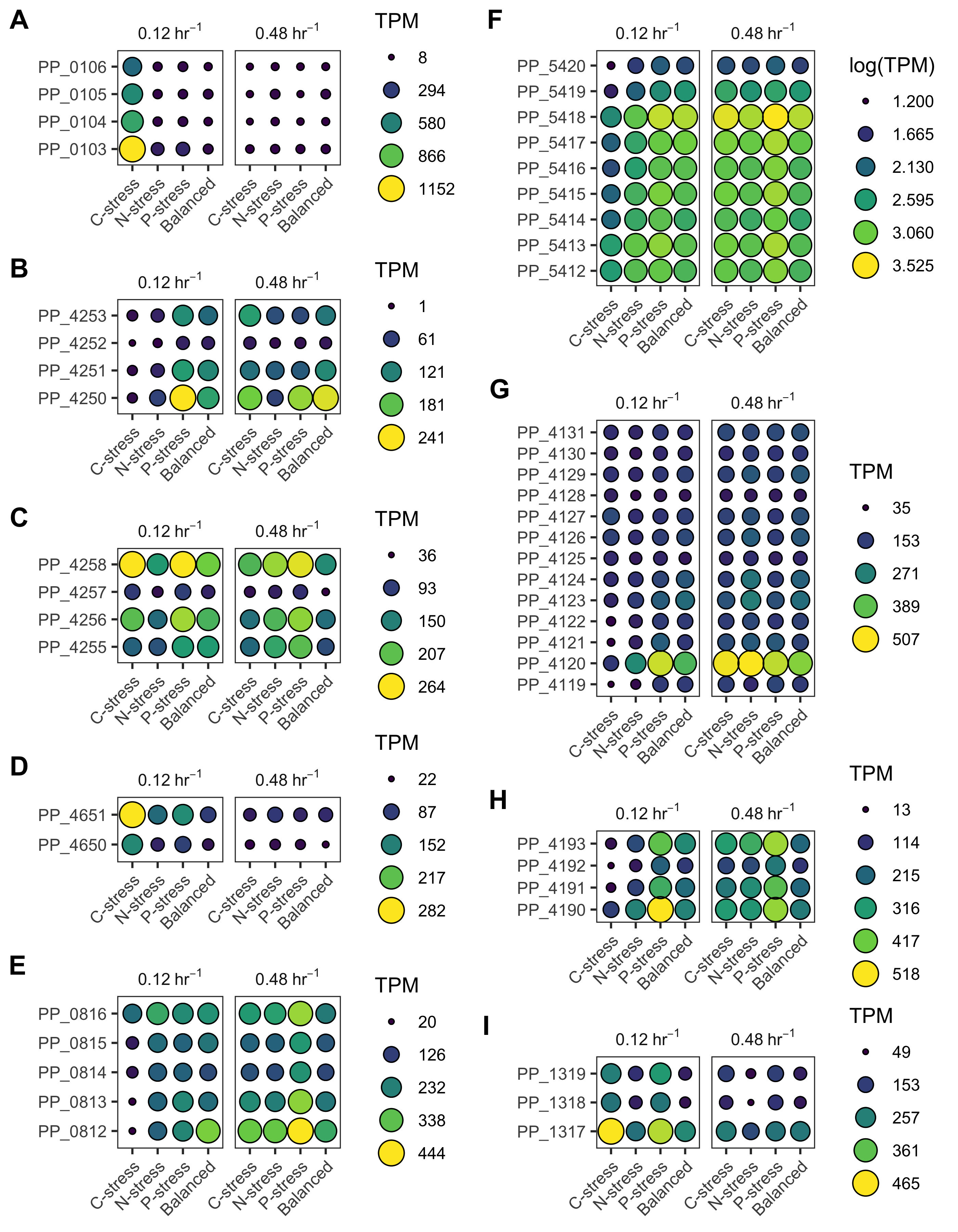


**Supplemental Figure 5**. Transcript expression of genes involved in the electron transport chain. Genes shown include terminal oxidase *aa_3_* (**A**), terminal oxidase *cbb_3_*-1 (**B**), terminal oxidase *cbb_3_*-2 (**C**), terminal oxidase *cio* (**D**), terminal oxidase *cyo* (**E**), ATP synthase (**F**), *nuo* dehydrogenase (**G**), succinate dehydrogenase (**H**), and cytochrome bc_1_ (**I**). TPM, transcripts per million. Note that scales are different between plots.

**Supplemental Figure 6**. While transcriptome composition tends to converge at fast growth, N-stressed (0.48 hr^-1^) and Balanced (0.12 hr^-1^ + 0.48 hr^-1^) cells express genes which are rare in most treatments. **A**) Ternary plot comparing average relative percent transcript expression between C-, N-, and P- stress resource ratios at 0.48 hr^-1^, where each light grey circle represents a gene. Different colored circles reflect genes of interest; green genes are involved in the TCA cycle, purple genes are involved in the EDEMP pathway, dark grey circles are terminal oxidases and the ATP synthase, blue circles are genes involved in the response to N-stress, brown circles are genes involved in the response to P-limitation. **B**) Expression of genes (TPM), within each treatment, that are differentially expressed in either Balanced (0.12 hr^-1^ + 0.48 hr^-1^) and N-stressed (0.48 hr^-1^) cells vs all other treatments. X axis, log2fold change; Y axis, transcripts per million. Genes are faceted by nutrient stress and colored based on their expression at either 0.12 hr^-1^ or 0.48 hr^-1^.


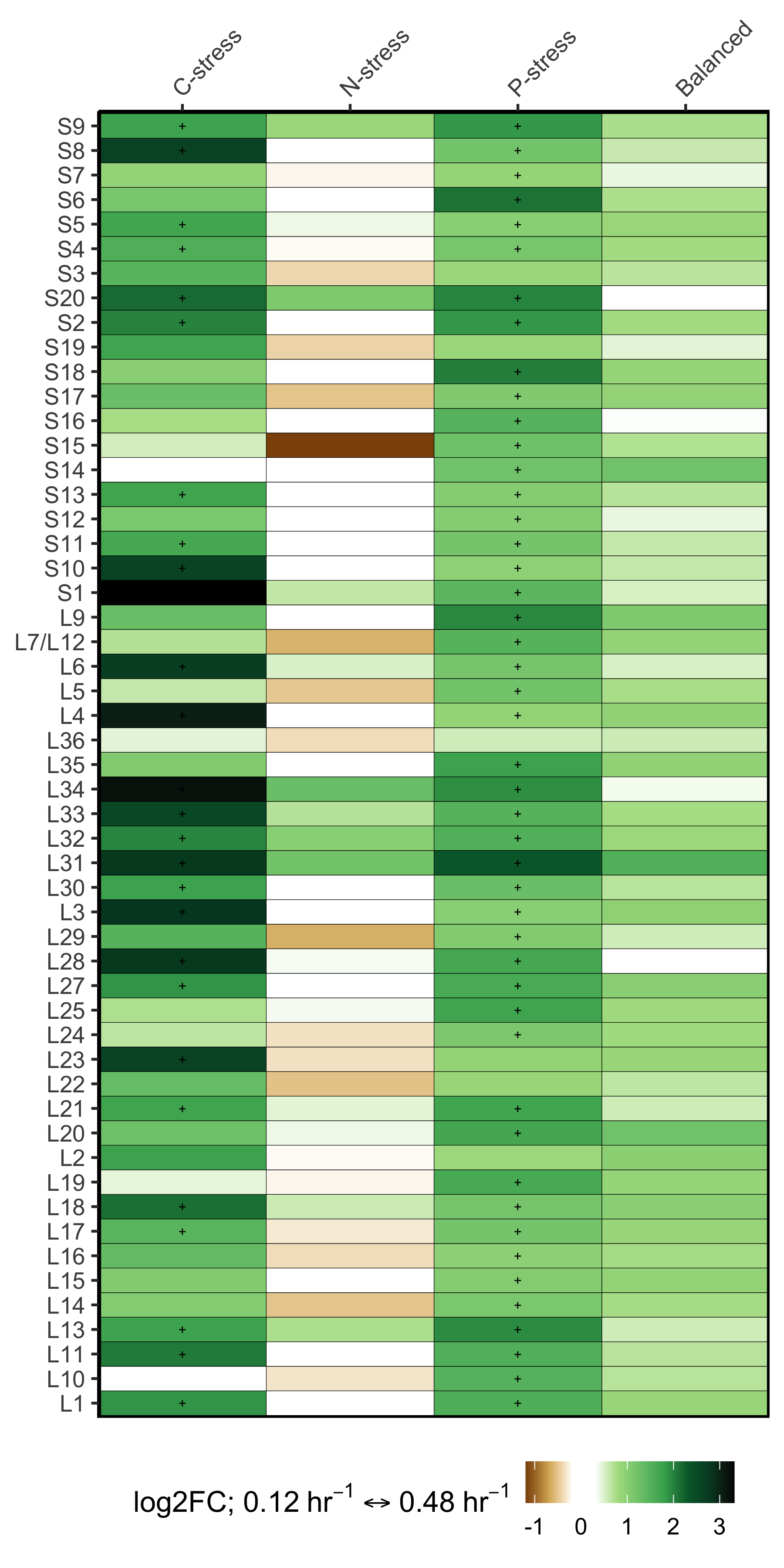


**Supplemental Figure 7**. Differential expression of ribosomal protein coding genes within each resource stress as a function of dilution rate. Significantly-different gene expression (p <0.05) is indicated by a plus sign. Log2FC; log2 fold change.
